# Horseshoe Kidney: A Cadaveric Case Report

**DOI:** 10.7759/cureus.81604

**Published:** 2025-04-02

**Authors:** Jacob Harwell, Kamal A Abouzaid, Ahmad Imam, Arpineh Petrosyan, Janan Niknam, Hannah Otten, Krithika Giresh, Sakshi A Gangodkar

**Affiliations:** 1 Medicine, William Carey University College of Osteopathic Medicine, Hattiesburg, USA; 2 Anatomical Sciences, William Carey University College of Osteopathic Medicine, Hattiesburg, USA; 3 Anatomy, William Carey University College of Osteopathic Medicine, Hattiesburg, USA

**Keywords:** cadaver case report, case report, clinical and functional anatomy, embryology to be correlated with gross anatomy, horseshoe kidneys, kidney, kidney function

## Abstract

This report describes a rare cadaveric case of a horseshoe kidney (HSK), a congenital anatomical anomaly characterized by fusion of the right and left kidneys. A distinct soft tissue band was identified joining the inferior poles of both kidneys, positioned inferior to the inferior mesenteric artery. The renal pelvises and ureters exhibited abnormal origin and course, which may increase the risk of compression and urinary tract obstruction. Additionally, accessory renal arteries supplying the inferior poles of the kidneys were identified and described. Measurements were taken to assess the dimensions of the connecting tissue band and the spatial relationship of the various relevant structures. This case highlights the rarity of the HSK and its associated anatomical variations and discusses potential implications such as urinary tract obstructions, infections, and kidney stone formation. A thorough understanding of these anomalies is critical for accurate diagnosis, effective management, and improving clinical outcomes in patients with HSKs. The literature reviewed in this case underscores the importance of recognizing such variations for better patient care and treatment strategies.

## Introduction

As one of the more common genitourinary congenital anomalies, a horseshoe kidney (HSK) is believed to have an incidence of every 1 in 400-1600 live births and is present in 0.25% of the general population, with a higher prevalence in men. The HSK is characterized by a connecting band of tissue that most commonly fuses the kidneys at their inferior poles. There are numerous hypotheses regarding the causation of the HSK, ranging across multiple medical etiologies. The embryogenesis involved in kidney development, final positioning, and blood supply is a complex and not well-understood process. Consequently, it is difficult to definitively point to a distinct event in embryogenesis that results in an HSK. Two leading theories attempt to explain the origins of this anomaly [1].

The most recent theory suggests that an HSK is caused by a teratogenic event that leads to abnormal migration of cells, forming a parenchymal isthmus between the kidneys [2]. This teratogenic effect may also help explain the increased risk of benign and malignant changes, including the development of renal cell carcinoma, transitional cell carcinoma (TCC), Wilm’s tumor, carcinoid, squamous cell carcinoma, and oncocytoma [3].

However, the classic theory of mechanical fusion suggests that during the metanephric stage (fourth week of gestation), the lower poles of the developing kidneys, while still in the pelvis and in close proximity to each other, come into contact and fuse at the midline with a fibrous isthmus [4]. This fusion is thought to occur due to abnormal flexion or growth of the developing spine and pelvic organs, leading to the fusion of the immature kidneys' nephrogenic blastemas. Normally, during the seventh and eighth weeks, the kidneys migrate out of the pelvis and rotate so that the renal pelvis faces medially [5]. In contrast, when an HSK ascends the isthmus, it becomes blocked and the HSK is malrotated, with each renal pelvis remaining anteriorly positioned at a lower lumbar level [4]. Frequently, the inferior mesenteric artery (IMA) is cited as being the physical barrier encountered by the isthmus of the HSK, although the isthmus is found caudad to the IMA in 40% of cases at the level of L4 [6].

The range of abnormalities associated with HSKs often results from malrotation of the kidneys, leading to repositioning of the ureters. This can cause the ureters to pass over the connecting renal band or descend along the ventral surfaces of the kidneys. While HSK typically shows no symptoms, there are occasional clinical manifestations. Pathological conditions are commonly observed, including ureteropelvic junction obstruction (UPJ) due to abnormal ureter insertion in the renal pelvis. Moreover, the presence of HSK anomaly may raise concerns about secondary renal pathology and potential malignancy of abnormal tissues [3].

Typically, an HSK is an incidental finding in patients undergoing evaluation for unrelated medical concerns. This report documents the gross anatomical findings of a case of an HSK observed during cadaveric dissection and discusses its potential clinical implications.

## Case presentation

An HSK was observed during the abdominal dissection of an embalmed 76-year-old female Caucasian cadaveric donor in the Ross Anatomy Lab at William Carey University College of Osteopathic Medicine. The cadaveric donor was obtained from the University of South Alabama Anatomical Gift Program.

**Figure 1:**
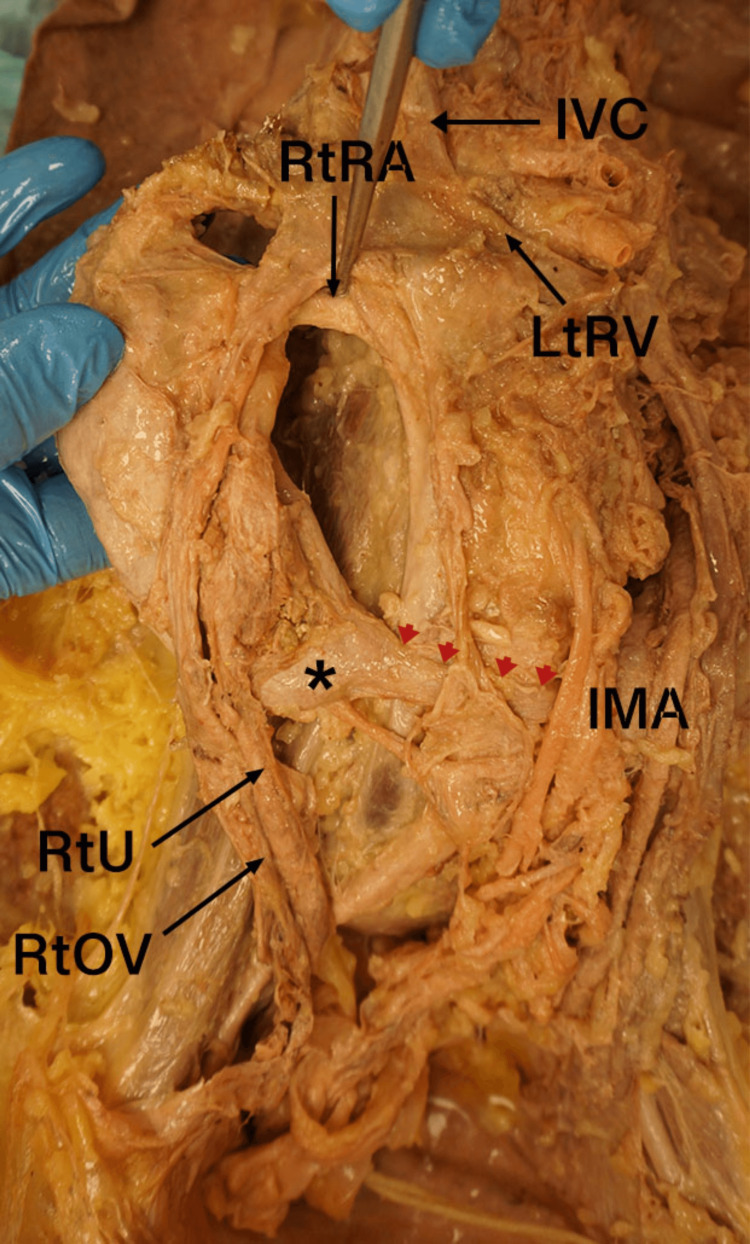
Anterior view of the posterior abdominal wall viscera demonstrating the HSK and the variant blood vessel RtRA: right renal artery; IMA: inferior mesenteric artery; RtU: right ureter; RtOV: right ovarian vein; arrow heads: isthmus; IVC: inferior vena cava; LtRV: left renal vein; *: lower pole of the right kidney

**Figure 2:**
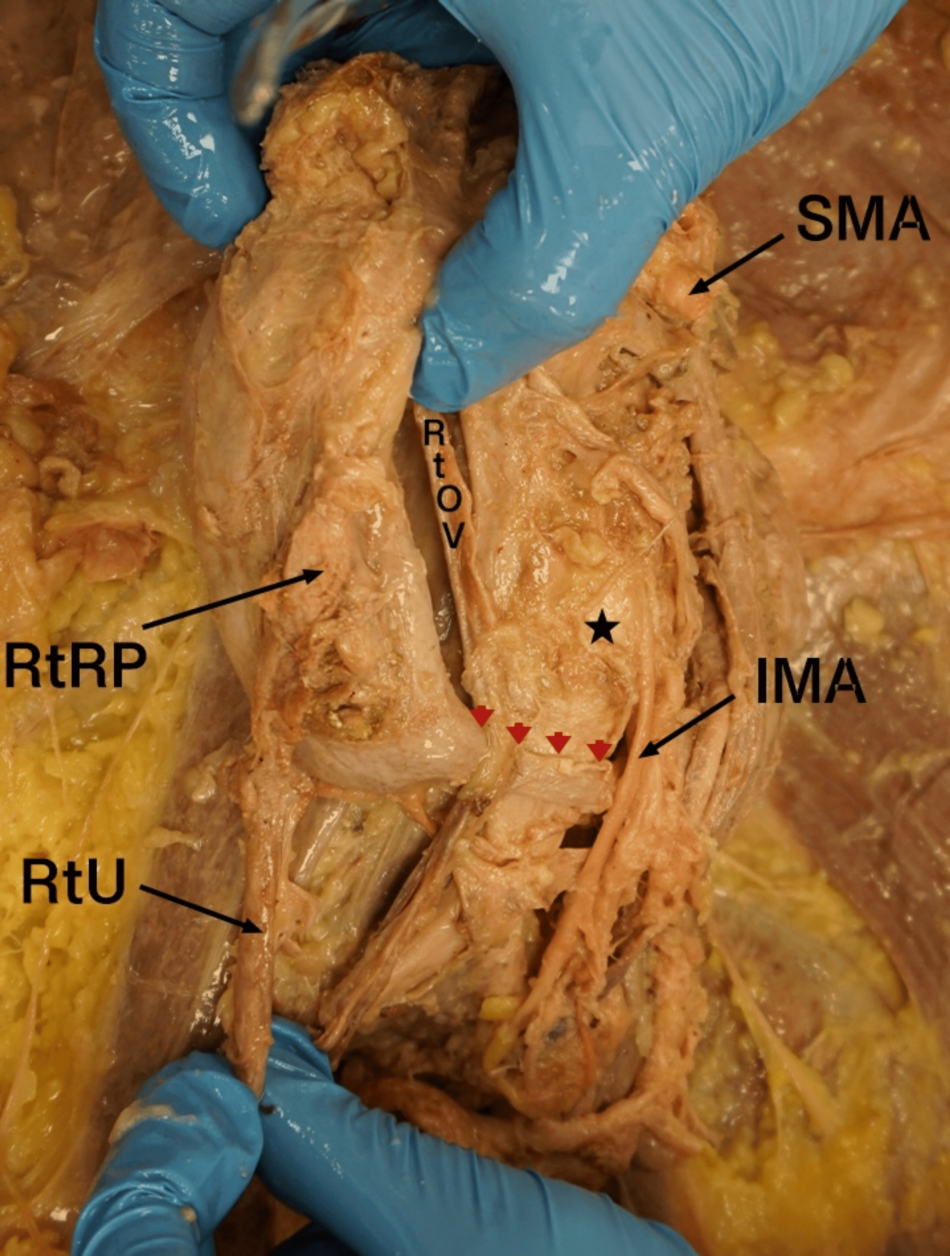
Anterior view of the right kidney demonstrating anterior positioning of the RtRP RtU: right ureter; RtRP: right renal pelvis; SMA: superior mesenteric artery; IMA: inferior mesenteric; RtOV: right ovarian vein; arrow heads: isthmus; star: abdominal aorta

During the dissection of the posterior abdominal wall viscera, a soft tissue band, isthmus, was noted connecting the inferior poles of both kidneys and positioned beneath the IMA (Figure 1). Additionally, both renal pelvises arose from atypical locations on their respective kidneys. The left renal pelvis emerged from the medial aspect of the left kidney at an abnormally inferior position, while the right renal pelvis originated from the anterior surface of the right kidney (Figure 2). Both ureters descended inferiorly over the isthmus and followed a normal course toward the urinary bladder.

**Figure 3:**
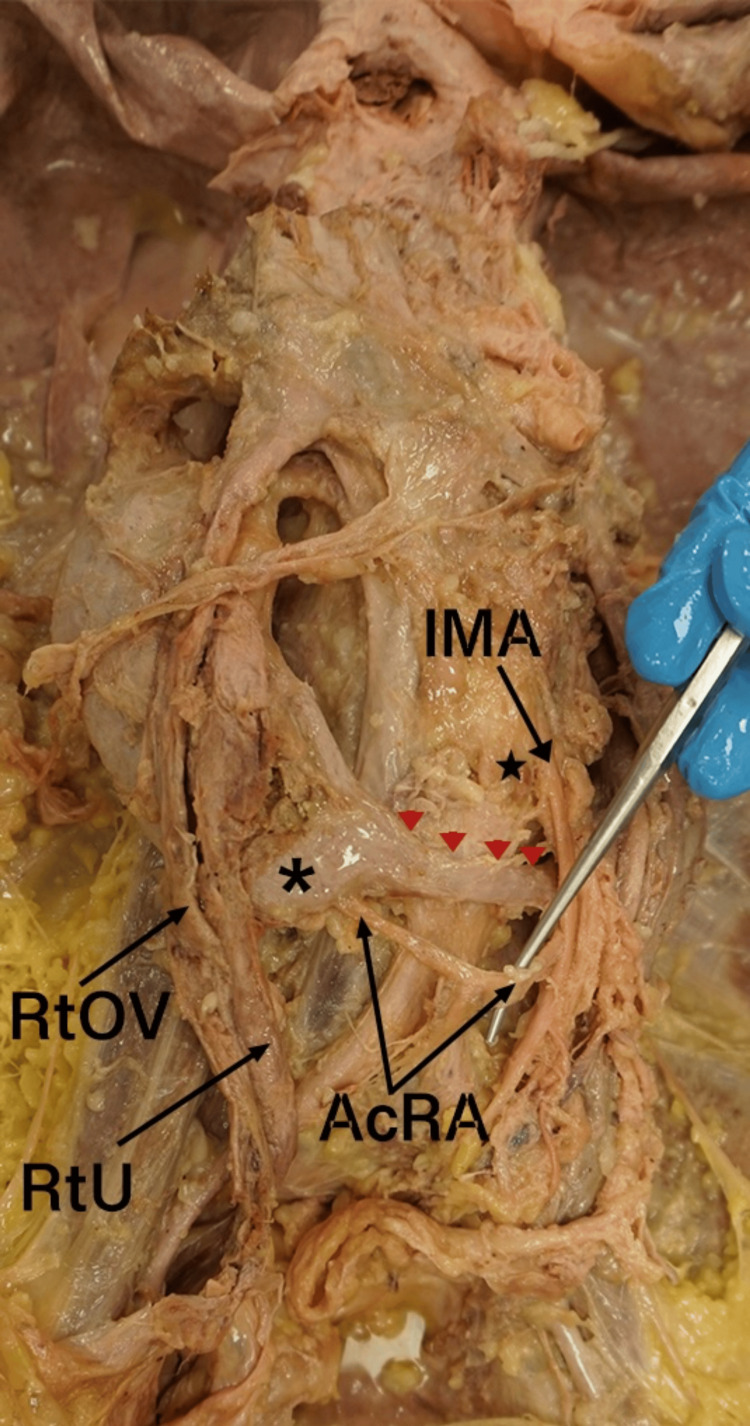
Anterior view of the HSK and AcRA anomaly IMA: inferior mesenteric artery; AcRA: accessory renal artery; *: inferior pole of right kidney; RtOV: right ovarian vein; RtU: right ureter; arrow heads: isthmus

**Figure 4:**
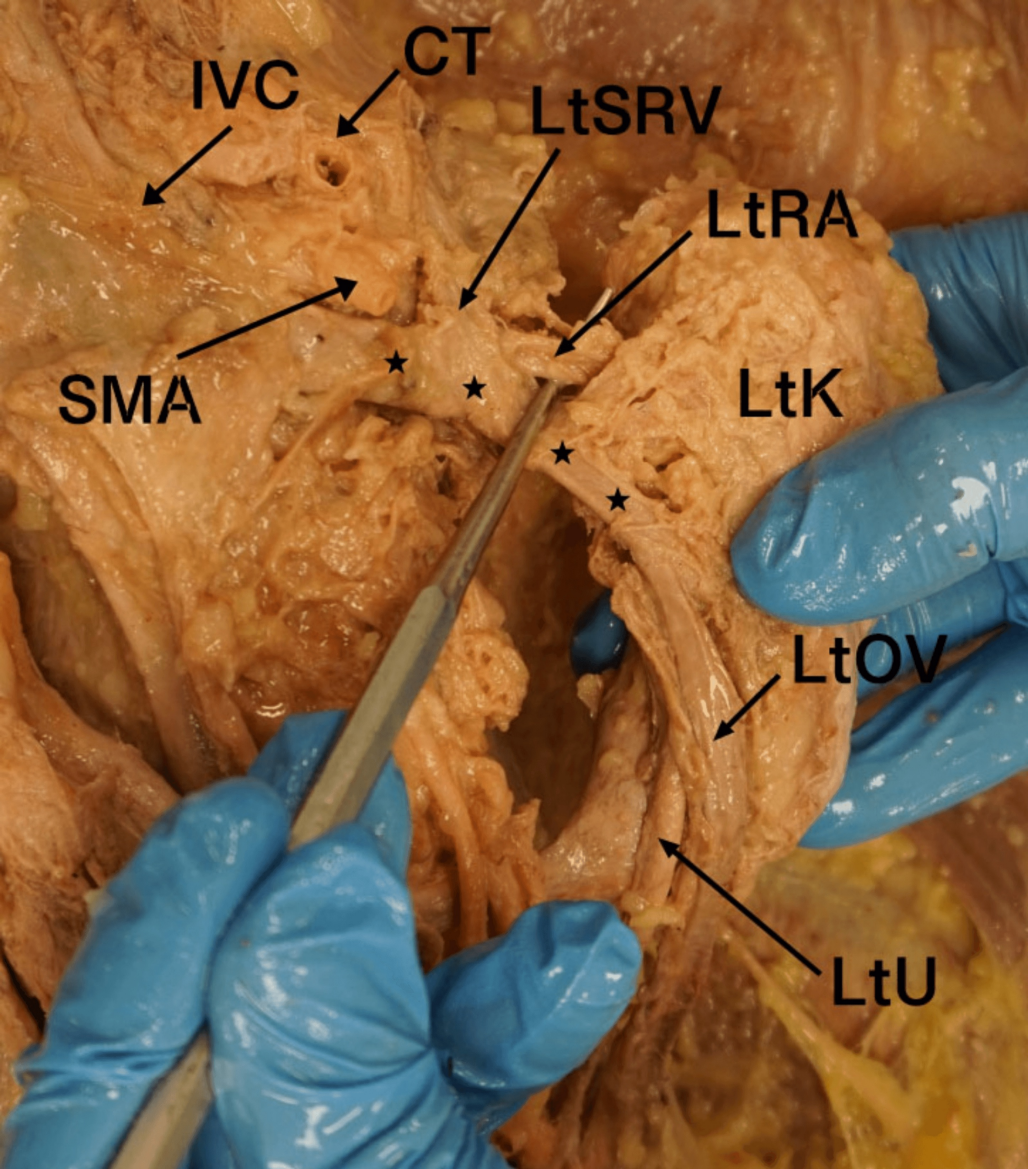
Anterior view of the left kidney showing the abnormal course of the LOV and LtRV SMA: superior mesenteric artery; CT: celiac trunk; LtSRV: left suprarenal vein; LtRA: left renal artery; LtK: left kidney; LtOV: left ovarian vein; LtU: left ureter; stars: left renal vein; IVC: inferior vena cava

Both renal arteries originated from the abdominal aorta and entered the medial aspects of the superior poles of both kidneys. In addition, both kidneys received an accessory renal artery (AcRA) originating from the median sacral artery below the level of the IMA. Each AcRA measured 29 mm in length before bifurcating into two distinct branches to supply the inferior pole of each kidney (Figure 3). The left suprarenal vein (LtSRV) demonstrated an unusual course due to the low-lying position of the left kidney. It ascended superiorly and then turned to the right to course under the superior mesenteric artery, ultimately draining into the inferior vena cava.  The right ovarian vein appeared to ascend along a typical path on the posterior abdominal wall. The junction of the left ovarian vein and the left renal vein was abnormally close to the level of the left renal pelvis (Figure 4).  

With the kidneys in situ, we took various morphometric measurements using a Marathon brand plastic vernier caliper. During the measurements, the kidneys and the arteries were in their discovered anatomical positioning, and no mechanical traction was applied to the tissue. The soft tissue band, the isthmus, measured 38.5mm from the inferior poles of both kidneys and 6mm in width at its widest point. The right renal pelvis originated 36mm inferior to the entrance point of the renal vessels, and the left was 34mm inferior to the same point but was positioned on the anterior surface. The ectopic position of the kidneys in the abdomen was determined based on the level of the vertebral column. In this case, the inferior edge of both kidneys was located at the level of the middle of vertebral body L5, indicating that it was lower in the abdomen than typically seen. 

This detailed anatomical description highlights the unique features of this HSK case and its associated variations, providing insight into the anatomical and clinical implications of this condition. 

## Discussion

Under normal circumstances, the kidneys are located in the retroperitoneal space on the posterior abdominal wall, typically positioned between the transverse processes of T12 and L3. The upper poles of the kidneys are generally positioned slightly medially and posteriorly compared to the lower poles. The right kidney is usually positioned slightly lower than the left kidney, likely due to the presence of the liver. Positioned superiorly and separated by renal fascia, the suprarenal glands (adrenal glands) lie on top of each kidney. Posteriorly, the diaphragm covers the upper third of each kidney, and the 12th rib passes over the upper pole of each kidney. At the medial border of each kidney is the renal hilum, where the renal artery enters, and the renal pelvis and vein exit the renal sinus. The renal vessels are anterior to the renal pelvis [7]. In our case, the lower edge of the lower poles of both kidneys was at the level of the vertebral body of L5, which indicates that the position of the kidneys was due most likely to the arrest of the connecting band by the origin of the inferior mesenteric artery, which prevented the ascent to the kidneys to their normal position. Moreover, both kidneys received blood supply from two sources: right and left RAs from the abdominal aorta that entered the medial aspect of the superior poles of right and left kidneys and AcRAs that originated from the median sacral artery, bilaterally. Each AcRA branched to supply the lower pole of each kidney. 

Accessory renal arteries are commonly found in HSKs and typically originate from different structures such as the abdominal aorta, common iliac, coeliac trunk, and superior mesenteric and inferior mesenteric arteries [8,9]. It is important to note that accessory renal arteries are end arteries, meaning that if the arteries are damaged, the part of the kidney they supply is at risk of becoming ischemic [9]. The main renal arteries usually develop normally. Additionally, the mesonephrogenic arteries often persist to provide blood supply to the upper pole of the kidney. In contrast, the inferior segmental metanephric arteries often persist and supply the lower pole of the kidney [8,10]. Their development is influenced by the functional and nutrient needs of the related organs during embryological vascular development [8]. The variation seen in our case is classified as a Type 2b arterial variation, meaning that the accessory artery arose from the sacral artery. In that study, this variation was the third most common and was seen in 9% of their subjects [10]. 

HSKs could result in UPJ obstruction, typically resulting from the elevated insertion of the ureter into the renal pelvis [3]. In our case, we observed that the renal pelvis of the right kidney was positioned on the anterior aspect of the kidney, and both kidneys had ureters that passed over the isthmus. These anatomical characteristics can lead to ureteral compression, causing urine stasis within the kidney and increasing the likelihood of stone formation, hydronephrosis, and infection. 

Individuals with HSKs have a higher risk of developing certain types of cancers compared to the general population. The most common cancer associated with HSKs is Wilms tumor, which primarily affects children. A retrospective study of 8,617 HSK patients found that 0.48% of them were diagnosed with Wilms tumor [11]. Another type of cancer that HSK patients are more susceptible to is TCC. The increased risk is due to factors such as chronic stasis, obstruction, infection, and stone formation in HSKs, which predispose individuals to the development of TCC [12]. 

The complexity and variability of the anatomical features associated with HSKs underline the critical importance of cadaveric studies in enhancing our understanding of this congenital anomaly. Through detailed anatomical exploration, cadaveric studies allow us to observe and document the unique vascular and ureteral configurations characteristic of HSKs. These findings are important not only in improving surgical outcomes by anticipating potential complications but also in providing insights into the developmental mechanisms underlying the HSK and its associated risks, such as malignancies and obstructions. 

## Conclusions

This case report emphasizes the critical importance of recognizing and understanding anatomical anomalies such as the HSK, as they provide valuable insight into the intricate development and function of the renal system. The ectopic positioning of the kidneys, abnormal courses of the renal arteries, the aberrant blood supply to the inferior poles of both kidneys, and the anterior positioning of the ureters highlight the anatomical complexity of the present case. A thorough understanding of these variations is essential for accurately diagnosing and managing patients with HSKs, as they may be prone to urinary tract obstructions, infections, stone formation, and malignancies. Continued investigation and documentation of such cases are vital for deepening medical professional understanding of rare renal anomalies, ultimately enhancing clinical outcomes and contributing to the advancement of patient care. 

## References

[1] Weizer AZ, Silverstein AD, Auge BK (2003). Determining the incidence of horseshoe kidney from radiographic data at a single institution. J Urol.

[2] De La Garza OT, Urest J, De La Vegan EU, Elizondo-Omaña RE, Guzmán-López S (2009). Anatomical study of the horseshoe kidney. Int J Morphol.

[3] Shah HU, Ojili V (2017). Multimodality imaging spectrum of complications of horseshoe kidney. Indian J Radiol Imaging.

[4] Abeshouse BS (1947). Crossed ectopia with fusion. Am J Surg.

[5] Natsis K, Piagkou M, Skotsimara A (2014). Horseshoe kidney: a review of anatomy and pathology. Surg Radiol Anat.

[6] Mouriquand P, Panait N (2012). Chapter 112 - renal fusions and ectopia. Pediatr Surg.

[7] Soriano RM, Penfold D, Leslie SW (2024). Anatomy, abdomen and pelvis: kidneys. StatPearls [Internet].

[8] Cocheteux B, Mounier-Vehier C, Gaxotte V (2001). Rare variations in renal anatomy and blood supply: CT appearances and embryological background. A pictorial essay. European Radiology.

[9] Londhe SS, Londhe B, Puranik MG (2024). Assessing incidence and variations of accessary renal arteries pertaining to origin, branching and patterns of segmentation: a cross sectional study. Walawalker Int Med J.

[10] Hekimoglu A, Ergun O, Birgi E, Turan A, Hekimoglu B (2023). Evaluation of renal artery variations in horseshoe kidneys with computed tomography. Urol Res Pract.

[11] Neville H, Ritchey ML, Shamberger RC, Haase G, Perlman S, Yoshioka T (2002). The occurrence of Wilms tumor in horseshoe kidneys: a report from the National Wilms Tumor Study Group (NWTSG). J Pediatr Surg.

[12] Balawender K, Cisek A, Cisek E, Orkisz S (2019). Anatomical and clinical aspects of horseshoe kidney: a review of the current literature. Int J Morphol.

